# The Value of Immersive Media in Expanding Chinese Public Cultural Participation and Its Realization Path From the Perspective of Cultural Education

**DOI:** 10.3389/fpsyg.2022.915913

**Published:** 2022-06-28

**Authors:** Wujin Cai, Yuan Liu

**Affiliations:** National Institute of Cultural Development, Wuhan University, Wuhan, China

**Keywords:** cultural education, immersive media, public cultural participation, cultural participation spaces, value

## Abstract

This paper mainly introduces the application of immersive media in Chinese public cultural participation from the perspective of cultural education, as well as the important value of the application of immersive media in expanding the breadth, accuracy, and depth of education and thereby improving the quality of education. On this basis, the realistic path of the role of immersive media technology in further realizing public cultural participation is discussed. First of all, through a questionnaire survey, it analyzes the problems existing in Chinese public cultural participation from the perspective of cultural education and the application of immersive media in Chinese public cultural participation. Secondly, from the aspects that the application of immersive media can extend the space of cultural participation, enrich the content of cultural participation and strengthen the value recognition of cultural participation, it demonstrates that immersive media helps to solve the dilemma of Chinese public cultural participation as a whole and thus improve the quality of education. This will bring new possibilities for expanding Chinese public cultural participation and promoting public social education and cultural quality education. Finally, it discusses the further development of immersive media technology in the field of cultural education from the aspects of optimizing the ecology of immersive media development, promoting the R&D and application of immersive media technology, and promoting the integration of immersive media with cultural participation space and cultural education content.

## Introduction

In the broadest sense, public cultural participation encompasses the activities and processes by which citizens enjoy public cultural services or participate in governmental public cultural decision-making and public cultural construction. Culture has important knowledge attributes, and public cultural participation can empower civic education. In this era, with the increasing status and role of culture in economic and social development and people’s lives, expanding public participation in culture so as to enhance the public’s cultural awareness, to raise the public’s sense of cultural subjectivity and subject presence, to strengthen the public’s recognition of national and ethnic culture, to improve the public’s cultural literacy and quality of life and integrate education into people’s lives in a culturally nourishing way. Particularly in a country like China, where cultural identity is the core of the basic social order, it is of great value to expand public cultural participation, effectively safeguard citizens’ cultural rights, enhance their sense of cultural acquisition and cultural confidence and promote the integration of their cultural life and education by working on the supply of basic public cultural services and the creation of a public cultural ecology. However, the current situation of public cultural participation in China is generally not very satisfactory, and the supporting role of modern information technology in public cultural participation has not been fully played.

The application and development of immersive media (IM) can maximize the close connection between public cultural venues and sites, as well as public cultural affairs and the public, reduce the psychological and cognitive distance between the public and public culture, and allow culture to be better integrated into citizens’ lives through media and medium and better play the educational function of culture, which includes such as 3D content, virtual reality, augmented reality, 360° images or videos. It becomes an inevitable choice to further highlight the role of immersive media in dealing with realistic social science problems and effectively enhance the cultural participation of the Chinese public.

## The Main Issues of Public Cultural Participation and the Application of Immersive Media in China From the Perspective of Cultural Education

### Data Description

The data in this article comes from the research activities of “Culture on the Front-line” organized by the National Institute of Cultural Development in Wuhan University in summer 2018. A total of 201 researchers were recruited and selected from college students to visit 28 provinces (including municipalities directly under the Central Government and autonomous regions) to investigate the current situation, demands and satisfaction of public participation in public cultural services through questionnaires. The study involved a total of 32,652 questionnaires from residents of a total of 28 provinces in China, including 10,398, 10,491, and 11,763 from the east, central and west, respectively, with 23,994 valid questionnaires and 73.48% valid questionnaire rate.

In this article, the valid questionnaires were statistically analyzed using SPSS 24.0 software, and the results are as follows. In terms of the gender structure of the participants, 44.9% were male and 55.0% were female. In terms of age, 17.8, 38.9, 23.9, 15.7, and 3.5% of each age group were 17 years old and below, 18–26 years old, 27–40 years old, 41–64 years old, and 65 years old and above, respectively. In terms of occupation, the majority of respondents were students, accounting for 44.4%. Employees of enterprises accounted for nearly one-fifth (18.7%). Civil servants and employees of public institutions, and freelancers accounted for 12.4 and 8.6%, respectively, fluctuating around 10%. Farmers accounted for the lowest percentage which was only 2.0%. Other self-employed businesses, retired people, temporary or unemployed and other occupations accounted for a small proportion of 13.7%.

In terms of education, junior college and bachelor’s degree groups are the most numerous, accounting for 50.3%, secondary and post-secondary education accounts for 40.8%, and the proportion of primary school and below and graduate and above education is smaller, at 3.3 and 5.4%, respectively. In terms of income, the proportion of non-income groups is the highest, accounting for 40.0%, the proportion of low-income groups (less than 3000 yuan) is 23.2%, the proportion of middle-income groups (from 3001 to 5000 yuan) and well-off groups (more than 5001 yuan) are 18.6 and 17.9%, respectively.

### The Main Problems of Public Cultural Participation in China

According to the investigation, with the continuous development of China’s public cultural service system and the increasing protection of cultural rights in China, the public cultural participation in China is increasing both in the breadth and depth, and the educational function of culture has a realistic foundation for further realization. However, there are certain problems in China’s public cultural participation, among which the following two aspects are particularly prominent.

#### The Overall Quality of Public Cultural Participation Is Not High

In recent years, as the construction of public cultural facilities in China has been increasing and the degree of public cultural participation has been improving. But, on the whole, the quality of public cultural participation in China is still not very high, which is mainly reflected in the low rate and frequency of public cultural participation, and it mainly focuses on enjoyment-oriented cultural participation, while construction-oriented cultural participation is relatively lagging behind.

**FIGURE 1:**
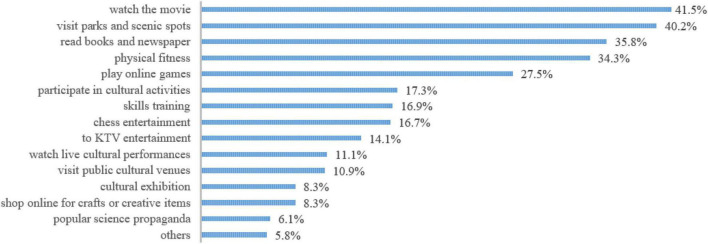
The main content of respondents participating in public cultural activities in China.

Specifically, the overall rate of public cultural participation in China is not very high, take Figure 1 as an example, and the participation rate for all types of public cultural activities is less than 50%. Moreover, the public culture participation is mainly based on leisure and entertainment-oriented public culture activities such as “watch the movie” (41.5%), “visit parks and scenic spots” (40.2%), “read books and newspaper” (35.8%) and (34.3%); The participation rate of knowledge-oriented public cultural activities, such as “popularizing science” (6.1%), “cultural exhibition” (8.3%), visiting public cultural venues (10.9%) and skill training (16.9%), is relatively low. At the same time, the frequency of participation in public cultural activities is also low. The frequency of participation in public cultural activities is mainly “once or twice a month” (35.0%), “once or twice a week” and “more than three times a week” account for 30.3 and 20.8%, respectively.

In addition, as the participation of Chinese citizens in public cultural policies and public cultural construction lags behind, the construction-oriented public cultural participation is far lagging behind the enjoyment-oriented public cultural participation (Cai, 2017), so public cultural participation is mainly manifested as citizens’ participation in public cultural activities and enjoyment of public cultural services.

#### There Is a Certain Imbalance in Public Cultural Participation

There is still obvious unevenness in public cultural participation in China between urban and rural areas, and between regions. Among them, there are certain gaps in the public cultural participation rate, frequency of public cultural participation, and public cultural participation environment among residents in east, central and west China. Correspondingly, there are also certain regional differences in the coverage of cultural education.

**FIGURE 2:**
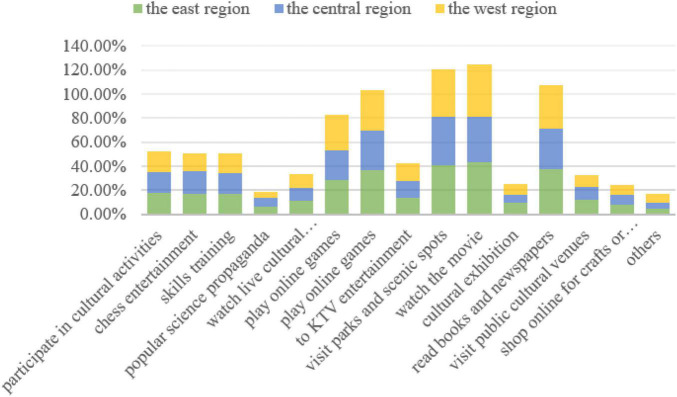
The participation rate of public cultural activities in east, central and west regions in China.

In terms of public cultural participation rates, the eastern regions basically have slightly higher participation rates than the central and western regions in both leisure and entertainment-oriented public cultural activities as well as educational and knowledge-oriented public cultural activities. As shown in Figure 2, in the activity of “visiting parks and scenic spots” with a higher overall public participation rate, the participation rate in the eastern region (40.7%) is higher than that in the central region (40.5%) and the western region (39.6%). The participation rate in activities such as “physical fitness” and “reading books and newspaper” is also significantly higher in the eastern region than in the central and western regions. Especially in terms of “cultural exhibition” and “visiting public cultural venues” etc., the participation status of the eastern region is significantly better than that of the central and western regions. For example, in the “cultural exhibition” activities, the eastern region (9.7%) is above the central region (6.7%) and the western region (8.7%). The eastern region (12.1%) is higher than the central region (10.9%) and western region (9.9%) in “visiting public cultural venues.”

**FIGURE 3:**
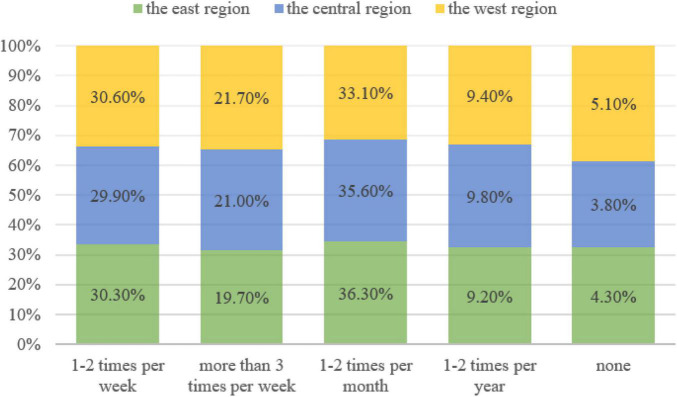
The frequency of public cultural participation in east, central and west regions in China.

In terms of the frequency of public cultural participation, as shown in Figure 3, “once or twice a month” is the highest, with 36.3% in the eastern region, 35.6% in the central region and 33.1% in the western region.

**FIGURE 4:**
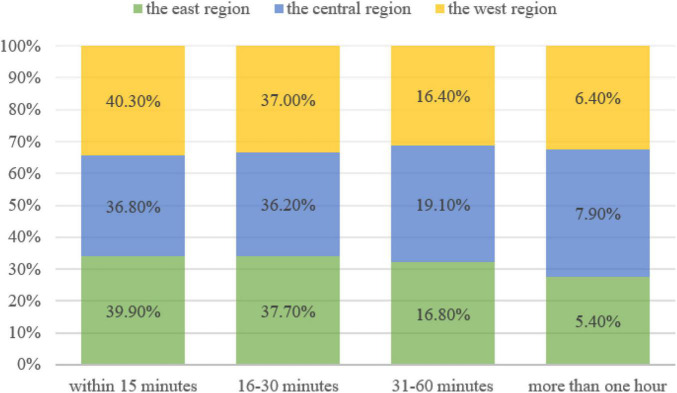
The public cultural participation environment in east, central and west regions in China.

In public cultural participation environment, as shown in Figure 4, 77.6% of respondents in the eastern region said they could get to public cultural facilities and venues in less than 30 min, higher than 73.0% in the central region and 77.3% in the western region. More than 90.0% of respondents in the East, Central, and West regions said they could reach the public cultural venues they regularly participate in within an hour. This shows that most current public cultural venues and facilities in China are relatively close to public living places, and it is quite convenient for the public to enjoy public cultural services, but relatively speaking, it is more convenient in the eastern regions e than in the central and western regions.

In other words, the coverage of cultural education in the eastern region is more extensive than that in the central and western regions, and it also has a better realistic foundation for further realizing the educational function of culture.

### The Application of Immersive Media in Chinese Public Cultural Services From the Perspective of Cultural Education

With the development of Internet technology, China has attached great importance to the integration of culture and online information technology, and has increasingly applied information technology in the construction of public cultural venues and facilities in an effort to provide high-quality public cultural services to the Chinese public. However, in terms of practical significance, the proportion of digital technology, especially immersive media, is still low in China’s public cultural venues and public cultural activities. However, the Chinese public has shown stronger expectations for the application of digital information technology and immersive media in the field of public cultural services.

Research data on the public’s demand for public cultural services shows that more than half (51.3%) of the surveyed people hoped local organizations to organize online cultural information lecture, and 59.0% of the public hoped local organizations to organize science and technology training. From the research data, we can see that in the current Internet information era, the Chinese public has a high demand for cultural technology and cultural information technology. Additionally, more and more people have placed expectations on media technologies that can enhance the sense of experience in the publicity, delivery and presentation of public cultural information.

However, in general, the current application of network information technology in public cultural services is not sufficient, and the application ratio of immersive media is even lower and it is only applied in some public cultural venues in some regions, which has greatly affected the current Chinese public’s satisfaction with public cultural services.

The mean value of respondents’ overall satisfaction with local public cultural services is 3.25 (with a maximum value of 5), as indicated by the Likert five scale statistics, which means that most of the public have low overall satisfaction with current public cultural services, and there is still a big gap between the current supply of public cultural services and public expectations and needs. Among them, 87.3% of the respondents said that the local public cultural services were already supported by technologies and contents such as e-reading, Internet and WiFi. But the mean value of the respondents’ satisfaction rating is 3.21 (with a maximum value of 5), which is not high. In addition, according to the results of the questionnaire research, most of the interviewed groups indicated that their local areas have been covered by public cultural service venues such as libraries, museums and art galleries, but the public’s satisfaction evaluation of these venues is not high.

For example, the satisfaction ratings of galleries and mass art centers (or cultural museums), and science and technology museums, and intangible cultural heritage exhibition are 2.68, 2.67, and 2.86, respectively, which are much lower than the overall satisfaction ratings of public cultural services. Actually, with the development of immersive media in China in recent years, public cultural venues such as museums, galleries, technology halls and intangible cultural heritage exhibition are exploring and expanding the application of immersive media in public cultural services which also reflects that the immersive media in China’s public cultural services has been attached importance and has great potential in expanding public cultural participation and responding to public cultural needs of the public.

As far as the practical significance is concerned, the widespread application of immersive media in the participation of Chinese public culture has a good practical basis. On the one hand, according to the China Internet (2021), China had 989 million internet users by the end of 2020, and the Internet penetration rate reached 70.4%. In particular, the total number of mobile internet users exceeded 1.6 billion; the number of 5G network users exceeded 160 million, accounting for 89% of the total number of 5G users worldwide (China Internet, 2021). The popularization of Internet provides a good technical basis for the application of immersive media. On the other hand, at present, many local governments and public cultural venues in China have actively explored the application of digital information technology and immersive media in the field of public culture, which provides a favorable practical basis for immersive media to widely serve the Chinese public’s cultural participation and promote the realization of lifelong education for all.

## The Value of Immersive Media in Expanding Public Cultural Participation From the Perspective of Cultural Education

Immersive media is an important medium for realizing the function of cultural education and an indispensable element of the knowledge dissemination system under the “Internet + Education” model, representing the trend of modernization of educational media. The application of immersive media helps to extend the space of cultural participation and thus expand the breadth of education, enrich the content of cultural participation and thus improve the accuracy of education, strengthen the value recognition of cultural participation and thus enhance the depth of education. And on this basis, immersive media helps to solve the dilemma of Chinese public cultural participation as a whole and thus improve the quality of education, so as to bring new possibilities for expanding Chinese public cultural participation and promoting public social education and cultural quality education.

### Immersive Media Helps to Extend the Space of Cultural Participation and Thus Expand the Breadth of Education

Public cultural participation must exist in a certain space, which is not only limited to physical spaces providing social education such as museums and libraries, but also may extend to institutional spaces that ensure citizens’ cultural participation. Only when citizens have a certain cognition of these cultural participation spaces, can the public demand for cultural participation be stimulated. And the value of immersive media is to allow the wider range of citizens to understand and approach cultural spaces that undertaking public education function more vividly, fully and distinctly through technical forms such as 3D and virtual reality, so that education is more widely available to more people and thus expand the breadth of education.

First of all, the traditional cultural participation space is the physical space for public cultural participation, which generally refers to the physical field composed of public cultural facilities, places and activities, etc. (Chen and Hou, 2017), and it is an important carrier of public cultural participation. Thus, the public cultural space equipped with immersive media is a realistic space of immersive scenes (Hu and Cheng, 2021).

Immersive media provides convenience for the public to understand the physical space of public cultural participation from the perspective of sensory experience, “enabling users to achieve immersive and resonant experience” (Yu and Zhang, 2020). The public cultural space represented by museums, libraries, cultural centers, etc., are rich in historical and cultural values. With the gradual application of emerging cultural communication forms such as VR, AR, mobile APP, and webcasting to the digital communication of public cultural spaces, the public can get a more personalized, immediate and ubiquitous sense of cultural experience, which is more effective in playing the educational and communication functions of public cultural space, and also better to highlight the historical and cultural values of cultural relics and artworks. In the immersive physical space, the combination of the audience’s senses with immersive experience can effectively narrow the distance between the public and the physical space, strengthen the meaningful emotional connection with the public, thereby attracting the public within the service area of the public cultural space to engage in daily cultural participation activities.

However, with the continuous development and improvement of immersive technology, its application can break through the constraints of time and space to a certain extent. The public cultural participation environment created by immersive media can break the limitations of traditional cultural participation in the integration of time and space, and create a digital virtual cultural space for the public that is not bound by space and time. With the support of immersive media, public cultural participation has the characteristics of “low threshold, openness, and virtuality” (Fu and Zhang, 2020), which enables the public to transcend the limitations of region, urban and rural, and time, reduces the economic and opportunity costs of public participation. Therefore, immersive public cultural participation can effectively make up for the current situation that public cultural participation in backward areas is far away in space and cultural activities are difficult to meet public demand, and to a certain extent can make up for the cultural education resources in backward areas and maximize the coverage of public cultural education. For example, immersive media can guarantee the institutional space of public cultural participation.

The public’s demand for cultural participation is not only reflected in physical space, but also reflected in the public’s desire to have an in-depth understanding of public cultural activity information, participate in the construction of public cultural service system and participate in public cultural decision-making through a normalized and institutionalized way. These needs are essentially the expectation of the public for the institutional space of public cultural participation. The application of immersive media is not only conducive to enhancing the public’s understanding of various institutional information and institutional ways of public cultural participation, but also can break through the restrictions of traditional channels of public cultural construction and public cultural decision-making system, and form a new, more convenient and accessible institutional platform and channel for public cultural participation. On the one hand, governments and public cultural institutions can more comprehensively and intuitively present various policies and information in the field of public culture through immersive media platforms. It also shows institutionalized ways and channels for the public to participate in public cultural decision-making, donate to public cultural venues, serve as public cultural service volunteers and other constructive matters. On the other hand, online media platforms and other immersive media can become innovative and institutional channels for regular communication, feedback and interaction between the government and the public, as well as between public cultural institutions and the public on public cultural decision-making and public cultural construction. The expansion from offline publicity and guidance to online active participation can enable the realization of a joint education mechanism for public participation in public cultural affairs, the expansion of institutional approach and space for public cultural participation, and the enhancement of the depth and quality of public cultural participation.

Education is both the process and the goal of public cultural participation. Today, “education is no longer the privilege of some distinguished talents or a prescribed activity for a certain age” (The International Education and Development Committee of UNESCO, 1996), but is increasingly oriented to include “the whole of society” (The International Education and Development Committee of UNESCO, 1996). In other words, we need to carry out Education For All, promoting the realization of the breadth of education. The international education field has proposed “Lifelong Education” (Lengrand, 1985) in the last century, from a spatial point of view, it breaks the traditional perception of education as school education and extends education to all aspects of society. Various types of immersive media applied to public cultural participation can gradually narrow the regional digital gap, develop non-formal education such as social education outside of school education through the information extension of cultural space, and finally promoting the inclusiveness of education, the fairness of education and the breadth of education.

### Immersive Media Helps to Enrich the Content of Cultural Participation and Thus Enhance the Accuracy of Education

Expanding the breadth of education lies in the extension of the space for cultural participation, while the core and root of enhancing the accuracy of education lies in the supply of attractive cultural participation content. Immersive media has become a new learning resource because of its immersive sensory experience (Liu et al., 2022), which has significant advantages in enhancing the cultural and educational value of public cultural content and gradually increasing public participation. The application of immersive media can enrich the content of cultural participation, provide personalized cultural services for different groups of the public, and innovate the public cultural participation model to make constructive public cultural participation possible, so that the public can be exposed to and aware of the cultural participation content to a certain extent, form their own understanding and interest in the corresponding content, inspire their own enthusiasm for cultural participation, and gain a good cultural education experience in the process of cultural participation.

Firstly, immersive media helps to provide personalized cultural engagement content.

Immersive technology has three main characteristics: immersion, interactivity and autonomy. Under the atmosphere created by immersive technology, audiences can feel immersive; at the same time, audiences can manipulate objects in virtual space and get certain feedback from human-computer interaction; in addition, audiences can stimulate their active learning interest and enthusiasm in the virtual or virtual-real environment on the basis of human-computer interaction. Immersive technology has distinctive advantages in designing and developing educational resources and forming personalized teaching methods. It can provide the public with suitable personalized learning situations according to their individual differences, and also provide them with suitable cultural participation contents according to their group differences, so as to effectively enhance learners’ individual feasible abilities and learning motivation, thus achieving the important goal of improving educational accuracy.

Immersive media helps to innovate content production methods and enrich the content of cultural participation while continuously integrating a large number of scientific and technological achievements. The combination of technology integration and content innovation has facilitated the emergence of immersive products of media education and promoted the science of immersive media education. Interactive and immersive technologies can make it easier and more convenient for students to access beneficial environmental settings, which has a broader appeal (Barton et al., 2020). In recent years, digital reading has shown significant advantages in enhancing the public’s cultural and aesthetic experience and strengthening the educational guidance function of works (Xia, 2017). For example, immersive technology combined with picture book education is applied to AR interactive picture books in public libraries and other cultural education spaces, allowing children to scan and present their doodles in three dimensions on smart terminals with animation and sound effects, stimulating children’s interest in the experience and improving their hands-on skills. Besides, the combination of immersive media and educational games, which integrating the educational reform concept of “teaching for fun” into cultural education, plays a role in knowledge skills teaching, simulating experiments and thinking training, and helps to enhance the effectiveness of cultural participation. In addition, immersive media can be used in distance education for people in remote and backward areas. In the cultural education for the public in remote and backward areas of central and western in China, a “cloud classroom” based on VR system can be created, where students can intuitively experience macroscopic or microscopic simulation scenes through VR headsets, at the same time, teachers can adjust classroom teaching activities through central control equipment. In the process, transmitters and receivers of knowledge can interact and communicate to make the perceptual experience of learners more realistic (ProQuest, 2019). The application of Head-Mounted Display Virtual Reality in Post-secondary Education and Skill Training brings convenient, engaging, and interactive choices to the traditional classroom environment, and provides additional capability over traditional methods (Concannon et al., 2019). This “two-way, real-time, interactive” (Zhao and Hao, 2018) form of education helps to enhance the effect of cultural participation, and alleviate the current problem of insufficient educational resources in remote and backward areas to a certain extent, realizing customized services for public cultural education.

Secondly, immersive media not only makes it more convenient for the public to enjoy public cultural services, but also innovates the mode of public cultural participation, promotes the public’s constructive cultural participation, thereby enriching the content of cultural participation and enhancing the public’s cultural participation experience in specific cultural education contexts.

As an important channel for the dissemination of public information and the formation of public issues, immersive media can play a role as a bridge linking private and public domains. With immersive media, it is possible to create a multidimensional, experiential educational context. In the virtual environment created by immersive media, the expression of public opinions breaks through the boundaries of space and time, forming a specific “public discourse space” (Habermas, 1989), and “realizes the extensive expression of individual opinions through peer-to-peer interactive communication” (Wang, 2019), improving the autonomy of the expression subject and expanding the influence of public opinion as well as the timeliness of cultural education. For example, with the development of fusion media, popular topics related to China’s “Sanxingdui Archeology” have formed public cultural issues in the fermentation of multiple platforms. This public cultural issue forms a synergy through TV media, short videos, small programs to realize information sharing among groups, build media communication matrix and form the cumulative effect among media. Then it has helped stimulate public discussion on key issues such as archeological excavation, cultural heritage protection and cultural relics activation and utilization.

At the same time, immersive media helps to promote active public participation in cultural building initiatives. As an important carrier for cultural communication and social education, immersive media can stimulate the public’s sense of self-perception and interactive sharing in the new scenarios it constructs, “integrating every individual’s knowledge, enthusiasm and wisdom into it and enabling people to realize sharing in the aggregation space with the largest individual choice” (Yu, 2007). Thus, it can maximize the promotion of people’s action on public cultural construction. At the same time, the creation of an educational environment for participation and sharing through immersive media is conducive to the cultivation and promotion of public cultural literacy and public spiritual realm, so as to more effectively drive the public consensus action on public cultural construction. Thus it can promote China’s current superficial public cultural participation to self-built shared cultural participation, management decision-making cultural participation and other deeper levels of cultural participation (Chen and Cui, 2017). The creation of such a cultural and educational environment is of great significance to the overall improvement of public cultural literacy and the enhancement of national cultural soft power.

### Immersive Media Helps to Strengthen the Value Recognition of Cultural Participation and Thus Enhance the Depth of Education

Public cultural participation is not only a cultural activity, but also an educational activity with characteristics such as equality and democracy, in which the public can reshape their spiritual world and enhance their cultural consciousness and cultural self-confidence. By carrying out rich public cultural education activities, public cultural venues and institutions can enhance the public’s cultural cognition and value judgment, further improve the social education function of culture, satisfy the public’s cultural desire, cultivate the public’s sentiment, enhance the public’s cultural identity, promote social fairness and harmony, and make the value of cultural participation deeply rooted in the hearts of the people, thereby enhancing the depth of education.

Firstly, public cultural participation contains inherent equality. Through immersive media, the public’s realistic feelings of cultural rights is enhanced.

Cultural rights refer to the rights and freedoms held by citizens, individually or collectively, to participate in cultural activities, to enjoy cultural resources and achievements, to share cultural benefits, and to promote cultural inheritance and development (Cai, 2014). Article 27 of the Universal Declaration of Human Rights clearly states that “Everyone has the right to freely take part in the cultural life of the society, to enjoy the arts and to share scientific progress and the benefits it produces” (The United Nations, 2008). This means that the core of cultural rights is to realize citizens’ equal participation and equal acquisition to cultural achievements and benefits, reflecting equality and fairness (Wang, 2015). The application of immersive media is to promote the equal and fair realization of cultural rights based on a broad guarantee of people’s right to cultural participation, thus better guaranteeing the fairness and equality of education. In the digital age with 5G technology, immersive media can expand the scope of the realization of cultural rights, enabling the public to break through the constraints of time and space, break down regional differences in the realization of cultural rights, enjoy public cultural services equally, and enjoy the fruits of cultural progress and innovation. For example, in recent years, more and more Chinese people can easily access cultural heritage and cultural scenes across the country by traveling to museums, art galleries and other educational public cultural venues on the cloud. They can also interact with public cultural venues and institutions, as well as with the public through live webcasts, messages, re-tweeting and discussions on online social media platforms.

At the same time, under the condition of immersive media technology, the public can participate equally in public cultural affairs and achieve freedom of cultural expression. Then the public can quickly respond to the expression and participation of the public through immersive media. In addition, the virtual space created by immersive media is a place for the expression of individual discourse, which reveals the inner world of the public in a unique way, hides and extends the public body, finds an ideal “cultural field” for the public, and promotes the expression and growth of civil rights awareness. Furthermore, in the process of application and experience of immersive media, the public will form a clearer, more intuitive and deeper cognition, feeling and understanding of cultural rights centered on participation. It is in the space and context combining virtual and reality that the public’s cultural rights can be more fully and comprehensively realized through various online or offline cultural participation activities, and the public can have a more practical experience of the equality of public cultural education.

Secondly, public cultural participation is democratic. With the help of immersive media, it is conductive to awakening the public’s subjective awareness of participating in public cultural decision-making.

In cultural engagement, the public is the subject of public cultural activities and affairs, and has the right to freely participate in various cultural activities, express their opinions and demands. Then they can influence and determine the direction of public cultural decision-making and the construction and development of public culture. In this process, the application and development of immersive media can construct the media space as a democratic platform for public participation, so as to promote the public from passive acceptance of public cultural services to active selection of public cultural service content and participation in public cultural decision-making. On traditional media platforms, elites and opinion leaders are more likely to “instill” their own values into the audience from top to bottom, which is essentially a neglect of the public’s right to express and know.

However, on the immersive media platforms represented by the Internet, the public has gained the opportunity to express themselves openly, and their right of discourse on cultural participation has been greatly enhanced. The right of discourse on public culture has shifted from “top-down” to “bottom-up,” and the public has a greater sense of participation in the expression of their opinions. The virtual spaces created by immersive media expands the field of current democratic practice. Web-based immersive media enables any organization or individual to express their positions, views and opinions freely through the Internet. As McLuhan points out, “What the mass media shows is not the size of the audience but the fact that everyone participates at the same time” (McLuhan, 2001).

Finally, public cultural participation can reshape the public’s spiritual world. Relying on immersive media, it helps to enhance the public’s cultural self-awareness and cultural self-confidence.

Cultural self-awareness and cultural self-confidence are the endogenous motivation of public cultural value identity. Immersive media has the function of knowledge dissemination and cultural education. Through its powerful communication advantages, immersive media can display and disseminate China’s excellent culture more comprehensively and deeply. It not only enhances Chinese people’s cognition and recognition of national and national culture, but also enhances their cultural awareness and cultural confidence.

In terms of reality, through the interactive display of immersive media, many “hidden” cultural relics in the museum come into the public’s view, realizing zero-distance interactive experience with the public, and enhancing the public’s awareness of cultural relics protection and inheritance of excellent traditional Chinese culture. The public’s recognition of the national tides and cultural museums is also gradually increasing. Nowadays, with the application of 5G and other new technologies, cultural variety shows such as National Treasure and China in Classic Books lead the public through the ancient and modern times and into the history by means of film-oriented expression and *trans-*temporal dialogue. The public has strengthened their cultural confidence in the process of “following dramas.” As well as the “Duanmen Digital Virtual Experience Hall” project by the National Palace Museum and the “Dunhuang Animation Drama” by the Dunhuang Research Institute, these projects showcase the charm of Chinese outstanding traditional culture in a three-dimensional manner, allowing the public to further participate in the preservation of cultural heritage while at the same time to enhance their confidence and trust in Chinese culture. In short, the application of immersive media has opened up new ways for the dissemination and display of Chinese culture, giving new impetus to the Chinese public’s cultural awareness and cultural confidence.

### Immersive Media Helps to Solve the Dilemma of Chinese Public Cultural Participation as a Whole and Thus Improve the Quality of Education

The sense of experience and presence constructed through immersive media can, to a certain extent, solve the current problem of low quality and uneven participation of Chinese public cultural participation in general.

On the one hand, the application of immersive media can break through the limitation of public participation time to a certain extent, allowing the public to use immersive media as a medium to experience and enjoy public cultural services at any time, which will help to greatly improve the proportion and frequency of public cultural participation. On the other hand, the application of immersive media can break through the limitation of space to a certain extent. Relying on immersive media, people enjoy public cultural services or participate in public cultural decision-making and commit to the construction of public cultural service system without necessarily going directly to the participating venues or being limited to the public cultural venues within the administrative areas. Instead, they can be “on-the-spot” anytime and anywhere through immersive media technology. In this way, a series of problems such as inconvenient venues, insufficient time, and inadequate construction of public cultural facilities can be solved to a certain extent in the process of public cultural participation, thus enhancing the degree of public cultural participation and enabling the public to receive the nourishment and edification of cultural education under a wider range of spatial and temporal conditions. This communication medium has innovated the way the public used to perceive and understand the world, and has had a significant impact on the current practice of socialized education.

The use of immersive media can promote the equitable allocation of social education resources and further strengthen the equity of education. The advantages of open and free cultural participation can greatly meet the public’s learning and education needs. On the other hand, immersive media encompasses almost all symbolic forms as well as new elements such as interaction and sharing, which greatly enhance the interest and attraction of education. Immersive media also provide conditions for lifelong education. In the era of knowledge economy, the in-depth integration of new media and the concept of lifelong education provides a broad development space for the public’s continuing education. Under this perspective of lifelong education, immersive media can reduce the cost of knowledge dissemination, and the public can be able to obtain educational resources with lower transaction costs and opportunity costs of participation, or even near zero costs. Public education of different regions, ages, and occupations will transcend the limitations of family education and school education, which is also in line with the future development direction of social education. So while cracking the dilemma of cultural participation, immersive media is also pushing the in-depth development of education, making it more three-dimensional and in-depth along with people’s cultural life.

## A Realistic Path to Further Enhance the Role of Immersive Media in Public Cultural Participation From the Perspective of Cultural Education

Immersive media has a profound value and role in expanding public cultural participation and enhancing cultural education functions in China, but its application in China’s cultural sector is still relatively short. It is necessary to work on optimizing the development ecology, applying technological innovation and promoting integration development, to further improve the value and role of immersive media in public participation in China.

### Optimize the Development Ecology of Immersive Media

The media ecological environment refers to the environment for the survival and development of mass media organizations (Wang, 2014), and the policy environment is crucial to the creation of a healthy development ecology for the media. After all, media policy affects the media ecology (Zhong, 2013). From the perspective of communication control analysis, as a disseminator of information, any mass media agency’ its communication behavior is always under the deep control of a social’s specific political systems, policies and regulations, and cultural paradigms (MBA Think Tank Encyclopedia, 2015). As a new direction of development and application in the context of media convergence, immersive media is highly valued and actively supported by Chinese government. The State Council of China has issued a series of policies to support the development of immersive media and industry.

In September 2020, the State Council of China issued the “*Opinions on Accelerating the Deep Integration of Media Development*” (CPGPRC, 2020a), which proposed to make good use of the achievements of information technology revolutions such as 5G, big data, cloud computing, Internet of Things, blockchain and artificial intelligence, strengthen the forward-looking research and application of new technologies, and promote independent innovation of key core technologies. In November 2020, the State Council of China issued the “*Opinions on Promoting the High-Quality Development of the Digital Cultural Industry*” (CPGPRC, 2020b), proposing the development of immersive business formats, guiding and supporting the application of technologies such as Virtual Reality (VR), Augmented Reality (AR), 5G+4K/8K ultra-high-definition in the cultural field. It also proposed to develop holographic interactive projection, night light show and other products, promote the transformation of existing cultural content into immersive content, and enrich virtual experience content. In addition, it supports cultural and cultural relic units to develop immersive experience projects with cultural resources and develop digital exhibition halls, online exhibitions and other services.

In addition, China’s “*14th Five-Year Plan and Outline of Long-Term Goals for 2035*” (CPGPRC, 2021) and the “*14th Five-Year National Informatization Plan*” (China Securities Journal, 2021). currently being formulated by China’s National Internet Information Office have all proposed to accelerate digital development. All of these policy documents aim to optimize the policy environment for the development of immersive media technology and provide good policy guidance and support for the application and development of immersive media.

However, there are still great deficiencies in the hierarchy, pertinence and systematicness of China’s policy support for immersive media. In order to further optimize the ecological environment for the development of immersive media in China, it is necessary to make efforts from the following aspects: On the one hand, we should further improve the level and strength of policy support for immersive media, especially introduce special laws and regulations to support the application and development of immersive media, support the research and development (R&D) of immersive media technology in the form of laws and regulations, and support the development of the application of immersive media technology in expanding public cultural participation. On the other hand, Chinese cultural authorities, network information management departments and other relevant departments should formulate (or jointly formulate) plans to promote the application and development of immersive media in the cultural field, and issue a series of targeted policies-based on the current development basis of immersive media and the needs of Chinese public cultural participation. In addition, we should establish and improve the fund to guide and support the development of immersive media in the cultural field, guide and support the construction and development of relevant enterprises and social organizations, and let social forces become a powerful force to promote the application and development of immersive media in the public cultural field.

### Promote the Research and Development and Innovative Application of Immersive Media Technology for Public Cultural Participation and Cultural Education

In terms of the current overall development status, immersive media technology in China is at an early stage of development, with both technical maturity and market maturity yet to be enhanced. In response to the need to expand public cultural participation in China, to meet the needs of building a public digital cultural service system and to achieve social education goals, it is necessary to promote the development and application of deep immersive media for public cultural participation in the following ways.

First, efforts should be made to develop new infrastructure for immersive media, as well as support hardware equipment to improve the performance of immersive media. The construction of key facilities and equipment should be promoted, especially the new basic design and equipment construction supporting 5G and VR technologies.

Taking VR technology as an example, from the perspective of hardware equipment, VR is similar to 3D, and you need to wear glasses to watch. The difference is that VR glasses are generally bulky and inconvenient to wear. If we can develop lightweight VR glasses without affecting the display effect, it will surely bring an upgrade of the experience effect for exhibitions in cultural and educational venue. As for 5G Technology, it needs the support of hardware equipment. With the large-scale deployment of 5G base station equipment in China, it is also necessary to develop cameras, game consoles and VR glasses that can support 5G network technology, so as to promote the wide application of 5G technology in public cultural venues, facilities and activities. In addition, there is an urgent need to accelerate the R&D and manufacturing of high-end cultural equipment related to laser playback, high-definition production and broadcasting, optical capture, performance special effects, performance interaction, and so on (Zeng, 2019).

Second, the research and development of related technologies and the production and innovation of related content should be strengthened. On the one hand, a high-quality immersive experience requires the support of multiple core technologies. The R&D of emerging technologies such as high-precision projection technology, sensing technology, 5G powerful transmission network, AI large-scale computing power, machine learning, intelligent robot, real-time 3D technology, and automation engineering should be strengthened. On the other hand, immersive content production in China is still in the experimental stage, without mature production methods and tools, making it difficult to produce (Ozgur et al., 2019). At present, it is time to break through the constraints of traditional content production methods and focus on improving the ability to tell stories and produce high-quality content in 360° environment. In addition, it is necessary to update and improve the immersive technology equipment and educational resources. China should vigorously support the R&D and investment of digital education resources, focus on the construction of information infrastructure in the central and western regions and other backward regions, and popularize mobile devices, so as to break the geographical time and space limitations, eliminate the “digital divide” and build an immersive digital education platform for all (Fu and Zhang, 2020).

Third, the innovative application of immersive media technology in public cultural participation as well as cultural education should be strengthened. In the future, with the mass popularization of 5G, deep immersive media is expected to see a full explosion in the near future. Therefore, the application of immersive media technology-related products and services in expanding public cultural participation and public education should be well planned, designed and developed. In particular, we should promote immersive media to link the cultural scenes in the social life of the Chinese people, respond to the problems and the expected direction of public cultural participation in China, promote more cultural participation and cultural education applications toward immersion and online, and create more immersive public cultural participation scenes that are closer to the needs of the people, more vivid and more diversified. Through the gradual cultural penetration from “form” to “content,” the educational function of culture is realized, and the spiritual guidance to the public is achieved from the superficial to the profound, so that the public can gain psychological pleasure and improve their inner cultural literacy through immersion experience.

### Promoting the Integration and Development of Immersive Media and Cultural Participation Space and Cultural Education Content

The key to giving full play to the value and role of immersive media in expanding public cultural participation lies in integrating immersive media technology into public cultural participation space and cultural education content, and becoming an integral part of public cultural space and cultural education content, and thus promoting the construction of the education system for all.

With the advent of 5G Internet era, cyberspace has dispelled the traditional mode of public cultural places, thus constantly changing the public’s lifestyle and cultural experience form. The emergence of immersive media is the necessity of the development of the information age. With the advent of the information age, public cultural places in the traditional sense can no longer meet the needs and expectations of the public. Only by closely combining public cultural places, public cultural education service content and emerging immersive media technology, can they meet the psychological needs of the public to the greatest extent and meet the needs of social education. Under the technical condition of 5G + AI + XR, it is possible for the public to be fully immersed and interact seamlessly in the narrative scene between the virtual world based on immersive media and the real physical environment, and all of these changes represent a step toward a more immersive, intelligent, and interconnected future.

To a large extent, this future is the full integration of immersive media technology and public cultural services. In terms of reality, China has made breakthroughs in big data, VR, AR, 5G, and other technologies, and has also made a series of integration explorations in the field of public culture.

In recent years, the rise of public culture in China, including “Cloud” performing arts, “Cloud” exhibition, “Cloud” concert, VR live broadcast, is the integration of immersive media technology and public cultural services preliminary results. However, this kind of integration still remains at the superficial level. Overall, the integration of immersive media and public cultural services, and the integration of the public’s cultural participation space and cultural education content, has broad prospects for development. It will gradually become an important field in the development of media technology and the construction of China’s public cultural service system, and will bring many unprecedented changes to the public’s psychology, cognition and behavior.

Therefore, there is still a need to continue to deeply expand the scope of immersive media and public cultural participation space and cultural education content integration, to strengthen the integration, to let history shine into reality, to interpret culture with technology, and to give new life to technology with culture.

With the efficient use of culture + technology, we will let immersive media technology support the construction of public cultural space, create public cultural space with innovative consciousness and artistic expression, and expand the application scope of immersive media in public cultural education places in Chinese cities. For example, we can learn from the experience of Montreal’s “Urban Memory” project, open up the scientific and technological boundaries of digital media, VR / AR, interactive devices, big data and smart tourism, and integrate the experience of cultural activities and site activation into the immersive experience segments of daily life. Urban public cultural space, as an important carrier of urban character and cultural self-confidence, represents regional identity and cultural values. The application of immersive media in public space can enhance the city image, convey the spirit of the place, create an immersive experience, and guide the public’s cultural participation behavior, so as to enhance the public’s sense of satisfaction and pleasure in cultural enjoyment activities and cultural construction actions, and realize the quality education and democratization education for the public in this process.

## Conclusion

In short, immersive media has great theoretical and practical significance for expanding Chinese public cultural participation, safeguarding people’s cultural rights, and promoting the integration of education into people’s lives in a culturally empowering way so that cultural education becomes an integral part of civic education. Although the current development of immersive media in China is still in its infancy, there are many difficulties in technology, cost, and content production that have not yet been broken through. However, standing on the cusp of 5G, culture is empowering immersive media technology, and immersive media technology is supporting the construction of China’s public cultural space, which can break through time, space and resource constraints. Thus, it provides the public with open, interactive and interesting public cultural experiences and services, bringing cultural education out of the constraints of traditional school education and classroom education, prompting the public’s knowledge acquisition and education improvement to shift from passive participation to active participation, from superficial participation to high-quality and deep participation in public cultural activities, and even making public cultural participation and cultural education a way of life for the Chinese people. Of course, there is still a long way to go in the application and development of immersive media technology in public cultural participation in China, and how to mobilize the enthusiasm of all parties and form a diversified development ecology is an important issue that needs to be further studied.

## Data Availability Statement

The original contributions presented in this study are included in the article/supplementary material, further inquiries can be directed to the corresponding author/s.

## Ethics Statement

Ethical review and approval was not required for the study on human participants in accordance with the local legislation and institutional requirements. Written informed consent from the (patients/participants or patients/participants legal guardian/next of kin) was not required to participate in this study in accordance with the national legislation and the institutional requirements.

## Author Contributions

WC: design the concept and outline, draft the main part of the manuscript, make important modifications to the manuscript, and approve the final manuscript to be published. YL: data collection and analysis and draft the part of the manuscript. Both authors contributed to the writing of the article.

## Conflict of Interest

The authors declare that the research was conducted in the absence of any commercial or financial relationships that could be construed as a potential conflict of interest.

## Publisher’s Note

All claims expressed in this article are solely those of the authors and do not necessarily represent those of their affiliated organizations, or those of the publisher, the editors and the reviewers. Any product that may be evaluated in this article, or claim that may be made by its manufacturer, is not guaranteed or endorsed by the publisher.
